# *Bacillus velezensis* BY6 Promotes Growth of Poplar and Improves Resistance Contributing to the Biocontrol of *Armillaria solidipes*

**DOI:** 10.3390/microorganisms10122472

**Published:** 2022-12-14

**Authors:** Ping Zhang, Guangqiang Xie, Lihai Wang, Yanqiu Xing

**Affiliations:** Heilongjiang Provincial Key Laboratory of Forest Sustainable Management and Environmental Microbial Engineering, Northeast Forestry University, Harbin 150040, China

**Keywords:** biological control, *Armillaria solidipes*, growth promotion, *Bacillus velezensis*, ISR, SAR, *Populus davidiana × Populus. alba var. pyramidalis* Louche

## Abstract

To improve the application of endophyte *Bacillus velezensis* BY6 from the xylem of poplar, the effect of BY6 on the growth of diseased *Populus davidiana* × *Populus. alba var. pyramidalis* Louche (Pdpap poplar) seedlings and the biological control effect on the pathogen *Armillaria solidipes* were tested using a plant split-root experiment. After applying BY6 to the roots of diseased Pdpap poplar seedlings, the results show that plant growth indicators (dry mass, fresh mass, and plant height) were significantly increased (*p* < 0.05), and genes related to auxin hormone signal transcription were activated. BY6 indicated a surprising control effect after the inoculation of diseased Pdpap poplar seedlings. Compared to the infected control group, the treated disease index of the diseased Pdpap poplar seedlings in the treatment group were reduced by 49.53% on the 20th day. The relative staining areas of diaminobenzidine (DAB) and Trypan blue decreased by 3.37 and 7.31 times, respectively. The physiological indicators (soluble sugar and protein) and oxidase indicators were significantly increased (*p* < 0.05). The expression levels of defense genes related to salicylic acid (SA) and jasmonic acid (JA) signaling pathways were significantly increased (*p* < 0.05). Amazingly, the results indicate that BY6 simultaneously activates induced systemic resistance (ISR) and systemic acquired resistance (SAR) in diseased Pdpap poplar seedlings and promotes growth. The results indicate that BY6 is a promising candidate for developing forest tree biofertilizers and biopesticides.

## 1. Introduction

The *Populus davidiana × Populus. alba var. pyramidalis* Louche (Pdpap poplar) on of the most commonly planted tree species in China. It is characterized by rapid growth, drought resistance, and low-temperature resistance [1,2]. Pdpap poplar is a female clone, and catkins can naturally fall off. It is also a model species for investigating woody plants [3]. With the continuous expansion of Pdpap poplar planting areas and the degradation of the environment, the incidence of root diseases caused by fungi is increasing, adversely affecting the growth of Pdpap poplar seedlings. Among them, Pdpap poplar *Armillaria* root rot (ARR) caused by *Armillaria solidipes* is one of the primary soil-borne diseases [4]. The chemical fungicides used to control the ARR of trees are of a single type, and the impact of use remains limited. It cannot meet the needs of the current nursery gardens. Some fungicides (methyl bromide) with better effects are prohibited from use because of environmental pollution [5,6]. Therefore, there is an urgent need to search for new green control agents for the ARR disease of Pdpap poplar.

Endophytes are often used to control root diseases in plants. Endophytes are a group of fungi and bacteria typically designated to colonize within the cells of healthy plants. Endophytes do not cause substantial damage to the host plant and establish a harmonious symbiotic relationship [7]. They can also inhibit various pathogenic fungi [8], promote plant growth [9], enhance plant tolerance to abiotic stress [10], and regulate and improve the soil environment [11]. Among these, *Bacillus velezensis* is an essential plant endophyte. Several studies have revealed that *B. velezensis* is a potent bacterium that produces lipopeptides (LPs), volatile compounds, and hydrolytic enzymes [12]. Huang et al. [13] isolated *B. velezensis* HYEB5-6 from boxwood leaves. They determined that this strain could significantly reduce the disease index of *Euonymus japonicus* anthracnoseon. Meng et al. [14] reported that a single strain of *B. velezensis* BAC03 could produce IAA, NH_3_, and ACC deaminase and promote the growth of nine crops. To date, the literature lacks a relevant report on BY6 strain inducing disease resistance and growth promotion of Pdpap poplar.

To survive, pathogens constantly break plant defenses. Several studies have shown that pathogenic bacteria can indirectly inhibit the disease-resistance signaling pathway mediated by salicylic acid (SA) or jasmonic acid (JA) to invade plants by regulating the ethylene (ET) content [15]. *A. solidipes* infects the Pdpap poplar after breaking through their defenses. Whether *B. velezensis* can induce the diseased Pdpap poplar to continue to defend the *A. solidipes* requires further in-depth understanding. In a previous study, we isolated and identified the bacterial strain *B. velezensis* BY6 from the Pdpap poplar xylem [16]. This study investigates the effects of BY6 on the growth, physiology, and disease resistance of diseased Pdpap poplar seedlings. We also determine the structure-activity relationship between the growth promotion and disease resistance of BY6 on diseased Pdpap poplar and provide a theoretical basis for future application of BY6 in forestry.

## 2. Materials and Methods

### 2.1. Source of Strains and Plant Material

Strain source: *A. solidipes* (number A2001) strain was isolated from diseased Pdpap poplar roots. A2001 strain internal transcribed spacer information was stored in National Center for Biotechnology Information (NCBI) GenBank, with the accession number OP787670.1. The *B. velezensis* BY6 was isolated from Pdpap poplar xylem. Its whole genome information has been stored in NCBI GenBank, the accession number CP051011-CP051012. The above strains were preserved at the Heilongjiang Provincial Key Laboratory of the Forest Sustainable Management and Environmental Microbial Engineering Department, Northeast Forestry University.

Plant Material: The experiments were conducted under controlled greenhouse conditions. Sterile Pdpap poplar seedlings used in this study were planted in a confined and sterile environment throughout the trial. After the stems were changed into seedlings, they were transferred to the rooting medium (½ Murashige and Skoog’s macronutrient (½ MS) define medium) at a temperature of 25 °C, with light for 16 h per day. After the Pdpap poplar grew 3~5 leaves, it was transplanted into the combined define culture medium of vermiculite + ½ MS. Before transplanting, 100 g of vermiculite was prepared and placed into flower pots (26 × 35 × 18 cm). A total of 50 mL of ½ MS define medium was added to each pot to ensure that the nutrient solution was evenly distributed in the soil and sterilized at 121 °C for 2 h. Each pot was watered with 50 mL of sterile water. The greenhouse temperature was maintained at 26 ± 1 °C and the relative humidity was 70 ± 2%, with a light period of 16 h and a dark period of 8 h.

### 2.2. Determination of Treated Disease Index and Relative Control Effect on Pdpap Poplar ARR after BY6 Induction

Healthy Pdpap poplar potted seedlings with a growth cycle of four weeks individuals were selected. The planted cultivar was highly susceptible to *A. solidipes*. The *A. solidipes* and BY6 were inoculated in a define PDA medium and cultured at 26 °C for 7 d. Hyphae from the edge of the colony were picked, transferred to a PD liquid medium, and rotary shaken (THZ-98A, Shanghai, China) at 160 rpm for 7 d at 28 °C. The concentration based on the ultraviolet spectrophotometer (Eppendorf D30, Berlin, Germany) OD_600_ reached approximately 1.9 × 10^8^–2.0 × 10^8^ CFU/mL.

After sterile Pdpap poplar seedlings were transplanted into vermiculite + ½ MS aseptic soil, the roots were washed and divided into two equal parts (part 1 and part 2), and then transplanted separately into two adjacent pots for 20 d. Seven days in advance, the treatment and infected control groups were inoculated with *A. solidipes* fermentation broth by root irrigation, and each pot was inoculated with 20 mL. After the Pdpap poplar leaves had obvious disease symptoms, BY6 fermentation broth (20 mL) was inoculated into each pot in the treatment group by irrigating the roots. An equal volume of sterile water was used as the control. Subsequently, the control and treatment groups were collected at 0, 6, 12, 24, and 48 h (from the 5th to the 8th leaf from the stem base), wrapped in tin foil, quickly frozen in liquid nitrogen, and stored at −80 °C.

Referring to the research method described by Hernandez et al. [17], based on the magnitude of 0~4, the disease degree of the aboveground leaves in the control and treatment groups at 0, 5, 10, 15 and 20 d was reported (leaf yellowing or wilting number/total leaf number, were 0 = 0%, 1 = 1~33%, 2 = 34~66%, 3 = 67~100%, 4 = dead plants). Referring to the research method described by Tang et al. [18], the treated disease index and control effect were calculated using the following formula: treated disease index = [∑ (number of disease-grade plants × representative value)/(total number of plants × representative value of highest disease grade)] × 100%, relative control effect = (control disease index- treated disease index)/(control disease index) × 100%.

### 2.3. Determination of Pdpap Poplar Enzyme Activity after BY6 Induction

To verify the BY6-induced resistance of Pdpap poplar, defense enzyme assays were performed on Pdpap poplar leaves of infection [18]. The leaves of the control and treatment groups at 0, 6, 12, 24, and 48 h (from the 5th to the 8th leaf from the stem base) were randomly mixed. They were then transferred to −20 °C in a cold mortar. After adding liquid nitrogen thoroughly grinding, the ground leaf tissue was transferred to a 1.5 mL centrifuge tube using a spatula. A solution of 0.02 M Phosphate buffer was added in a ratio of 1:9 weight of leave tissue to volume phosphate buffer. The mixture was vortexed for 1 min, followed by centrifugation at 3000 rpm for 4 min at 4 °C. A total of 50 μL of the supernatant was transferred into a 5 mL centrifuge tube. According into the kit’s (NJJCBIO, Nanjing, China) instruction manual, the reaction reagents were introduced to the control and measurement tubes and mixed. The absorbance OD value was measured (Eppendorf D30, Berlin, Germany), and the enzyme activity was calculated according to the following formula.

### 2.4. Detection of Reactive Oxygen Species and Cell Death in Pdpap Poplar Leaves after BY6 Induction

The experiment was terminated 20 d post-inoculation with the BY6 strain. Referring to the method described by Lin et al. [19], the leaves were immersed in diaminobenzidine (DAB) (Sigma-Aldrich D8001, Berlin, Germany) staining solution (1% (*w*/*v*)) for 50 min. They were then removed from the solution and placed in a mixture of 75% ethanol, 5% glycerol, and 20% distilled water. They tubes were incubated in a water bath (CU-600, Shanghai, China) at 100 °C for 30 min. The stained leaves turned brown, suggesting the production of H_2_O_2_. Referring to the method described by Hung et al. [20], the leaves were immersed in trypan blue (1% (*w/v*)) (Sigma-Aldrich V900876, Berlin, Germany) for 50 min, removed, and then placed in a centrifuge tube containing a mixture of 75% ethanol, 5% glycerol, and 20% distilled water. They were then placed in a water bath at 100 °C for 30 min. The staining result turned blue, suggesting that the Pdpap poplar leaf cells were necrotic.

### 2.5. Determination of Pdpap Poplar Seedling Growth after BY6 Induction

Referring to the method described by Goicoechea et al. [21], the whole Pdpap poplar seedlings were taken out. The root soil was washed with clean water, placed in water, and then straightened. Vernier calipers (SATA91511, Shanghai, China) were used to measure root length and plant height. Plant height is defined as the distance from the ground position to the tip of the tallest leaf. The whole Pdpap poplar was placed in a sealed bag filled with distilled water to ensure that the entire seedling could fully absorb water. It was soaked in water for 24 h, and the fresh mass was measured after removing the excess water with filter paper. The whole Pdpap poplar seedling was cut into pieces for dry matter quality determination and placed in a glass petri plate. It was then placed in a blast drying oven (BPG-9206A, Shanghai, China) at 115 ± 10 °C for 30 min. The Pdpap poplar seedling was naturally cooled, placed in an oven at 65 °C for 4 h, and weighed using an electronic balance (Shimadzu ATX224R, Kyoto, Japan).

### 2.6. Determination of Soluble Sugar and Soluble Protein in Pdpap Poplar Seedlings after BY6 Induction

One gram of leaf tissue was washed with 0.9% normal saline to the grind. The Phenol and Coomassie brilliant blue G250 methods [21] were used to determine the soluble sugar and protein, respectively. The Pdpap poplar leaves were cut and placed in a 50 mL centrifuge tube. Five mL of deionized water was added to a water bath at 100 °C for 30 min. The tissue fluid was filtered through filter paper and diluted five times, yielding a total of 0.5 mL of the dilution. A total of 200 mg of Pdpap poplar leaves was used similarly. Five mL of deionized water was added to the ground sample, and the homogenate was centrifuged at 8000 rpm for 5 min (Eppendorf D30, Berlin, Germany). The supernatant was diluted five times with deionized water, and then 0.1 mL of the dilution was transferred to a centrifuge tube to determine soluble protein content in the Pdpap poplar. Simultaneously, a standard curve was prepared using bovine serum albumin (BSA) as the standard. Five mL of Coomassie Brilliant blue G250 was added to the standard and sample test tubes. It was inverted, mixed several times, and maintained for 5 min. A 1 mL was extracted and put in a micro cuvette. Absorbance was measured at a wavelength of 595 nm. The soluble protein content was then calculated.

### 2.7. Determination of Disease Resistance and Auxin Gene Expression in Pdpap Poplar after BY6 Induction

Disease resistance and growth-related gene expression in Pdpap poplar were determined after BY6 induction. The leaves of the control and treatment groups at 0, 6, 12, 24, and 48 h (counting from the 5th to the 8th leaf from the stem base) were randomly mixed. They were then transferred to −20 °C in a cold mortar. After adding liquid nitrogen thoroughly grinding, the ground leaf tissue was transferred to a 1.5 mL centrifuge tube using a spatula. Total RNA was extracted using RNA Extraction Kit (BIOTEKE PR3502, Beijing, China). The extracted RNA was assayed for RNA concentration using a UV spectrophotometer (BIOTEKE ND5000, Beijing, China) (OD260/280). The RNA final concentration of 1 ug, and cDNA was synthesized using a PrimeScript^TM^ RT reagent Kit with gDNA Eraser (Takara, Tokyo, Japan).

Primers were designed using the Primer 5.0 online tools (https://www.premierbiosoft.com/primerdesign/index.html accessed on 26 October 2022), and were the primers synthesized by Shanghai Sangon Bioengineering Co., Ltd (Shanghai, China). *Act* (P)-L and *Tub* (P)-L were housekeeping genes. The sequences of housekeeping genes and PCR primers are illustrated in (Table 1). PCR reagents (SYBR Green Realtime PCR Master Mix 10 μL (Takara RR820A, Tokyo, Japan), cDNA 2 μL, Primer F (10 μM) 1 μL, Primer R (10 μM) 1 μL, and ddH_2_O 6 μL) were added. The RT-qPCR (Agilent Mx3000P, Calif, USA) reaction program was as follows: (denaturation: at 94 °C, for 10 s, annealing at 60 °C, for 30 s, extension at 72 °C for 30 s, 30 cycles). The relative expression levels of the genes obtained by the reaction were calculated using the 2^−ΔΔCT^ method [22]. Four biological replicates were used for each gene.

### 2.8. Statistical Analysis

All experimental data were subjected to analysis of variance (ANOVA) using the SPSS software v19.0 (IBM Corp., Armonk, NY, USA). The statistical significance of the difference between means was determined using Duncan’s multiple-range test and an independent-sample T test (*p* < 0.05).

## 3. Results

### 3.1. Treated Disease Index and Relative Control on Pdpap Poplar Root Rot after BY6 Induction

**Figure 1:**
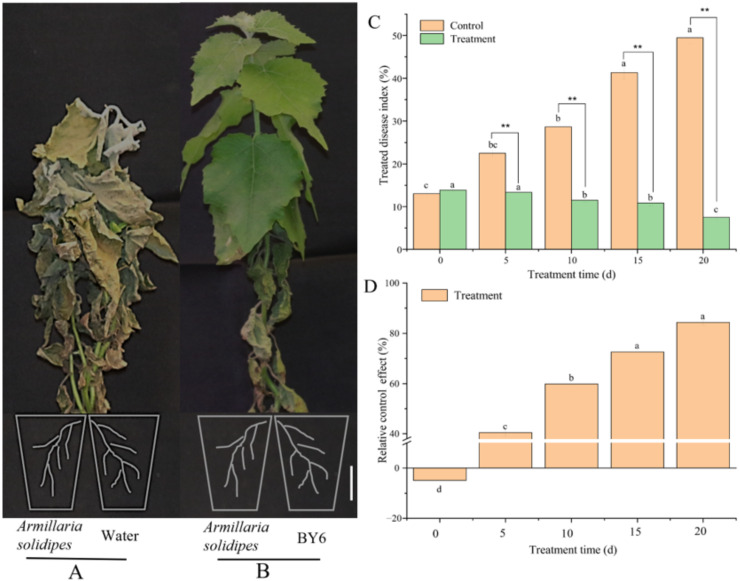
Pdpap poplar ARR symptoms and treated disease index. (**A**): Water control; (**B**): Inoculated BY6; (**A**,**B**): the inoculation of *A. solidipes* in advance 7 d, (**C**): the treated disease index; (**D**): Relative control effect; The roots of Pdpap poplar seedlings were divided into two equal parts (part 1 and part 2), and transplanted separately into two adjacent pots. Architect scale is 1 cm; The experiment was carried out in four biological replicates; ** indicate significant differences between the groups at the same time point (ANOVA, ** < 0.01), and the different lowercase letters indicate significant differences for the same treatment group at different times (ANOVA, *p* < 0.05).

On the 20th day after inoculation with *A. solidipes*, Pdpap poplar seedlings in the greenhouse demonstrated disease symptoms similar to those in the field. The leaves in the infected control group revealed yellowing and withering and then began to die one after another (Figure 1A). On day 20, the treated disease index reached 49.53%, an increase of 36.51% compared with 0 d (Figure 1C). However, after BY6 induction, only the leaves below the ground demonstrated wilting symptoms, suggesting that the disease symptoms of the plant were relieved. New leaves were successively grown (Figure 1B). On day 20, the treated disease index decreased by 42.01% compared to the infected control group, and the relative control effect indicated an upward trend. The control effect on the 20th day reached 84.31%, an increase of 89.22% compared to that at 0 day (Figure 1D). This result indicated that the diseased Pdpap poplar seedlings’ disease index was significantly reduced after BY6 induction and had a certain biological control effect.

### 3.2. Effects of BY6 Induction on the Activity of Defense Enzymes in Pdpap Poplar

**Figure 2:**
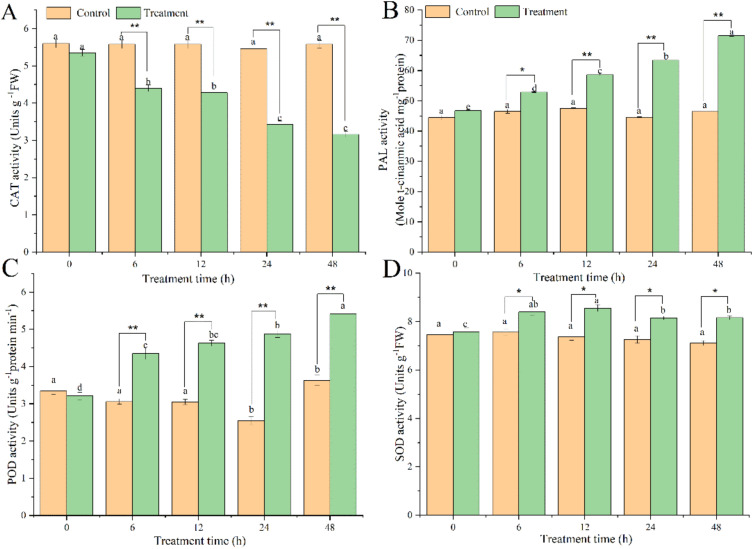
The effect of BY6 on Pdpap poplar oxidase indicators activity. (**A**): Catalase (CAT). (**B**): Phenylalanine ammonia-lyase (PAL). (**C**): Peroxidase (POD). (**D**): Superoxide dismutase (SOD). The experiment was carried out in four biological replicates. The graphical values were the average of four biological repeats. Error bars represent standard errors of the mean (*n* = 4) at each time point. Different * indicate significant differences between the groups at the same time point (ANOVA, * < 0.05; ** < 0.01), and the different lowercase letters indicate significant differences for the same treatment group at different times (ANOVA, *p* < 0.05).

According to the results (Figure 2A), Pdpap poplar catalase (CAT) activity in the treatment group exhibited a downward trend, from 0 to 48 h. It reached a minimum value at 48 h, which was 0.59 times lower than that at 0 h, while the difference between the infected control groups was insignificant. Phenylalanine ammonia-lyase (PAL) activity treatment group demonstrated an upward trend from 0 to 48 h (Figure 2B), reaching a maximum value at 48 h, which was 1.52 times higher than that at 0 h. In contrast, the differences between the values of the infected control groups at different points of time were insignificant. These results indicate that BY6-induced diseased Pdpap poplar decreased CAT activity, and increased PAL activity.

Peroxidase (POD) enzyme activity treatment results indicated that the treatment group had an upward trend from 0 h to 48 h (Figure 2C) and reached a maximum at 48 h. Compared with 0 h, the enzyme activity increased by 1.68 times. The infected control group was insignificant, and only a small increase was observed from 24 to 48 h. The superoxide dismutase (SOD) enzyme activity treatment group increased from 0 to 12 h (Figure 2D), reached the maximum value at 12 h, and increased by a factor of 1.12 compared to that at 0 h. It then stabilized from 24 to 48 h, while the difference between the groups was insignificant; this indicates that BY6 induced Pdpap poplar seedlings, increased POD and SOD activities in Pdpap poplar.

### 3.3. Staining Results of Reactive Oxygen Species and Cell Death in Pdpap Poplar Leaves after BY6 Induction

**Figure 3:**
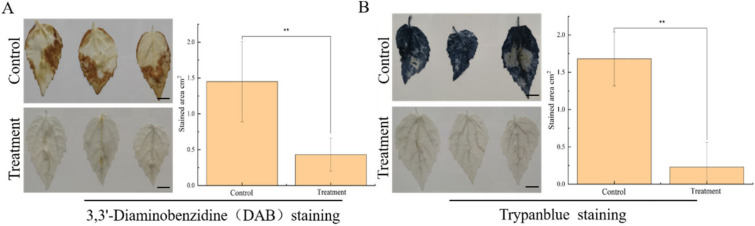
DAB and Trypan blue staining result. The results of Pdpap poplar leaves with DAB (**A**) and Trypan blue (**B**) 20 days after inoculation of treatment statistical analysis of DAB and trypan blue using Image J software; Biology repeated three times; ** indicate significant differences between the groups (ANOVA, ** < 0.01); Architect’s scale is 1 cm.

On the 20th day after the infection experiment, the Pdpap poplar leaves were stained with DAB and trypan blue, respectively. The results shown in Figure 3A that the DAB staining treatment and infected control groups had different degrees of staining, and the relative staining area of the infected control group was 3.37 times higher than that of the treatment group. The results shown in Figure 3B were obtained using trypan blue staining, but the relative staining area of the infected control group was 7.31 times higher than that of the treatment group. Both indicate that BY6 induction significantly reduced the reactive oxygen species content and the degree of necrosis in the leaf cells of diseased Pdpap poplar.

### 3.4. Effects of BY6 Induction on Pdpap Poplar Growth

**Figure 4:**
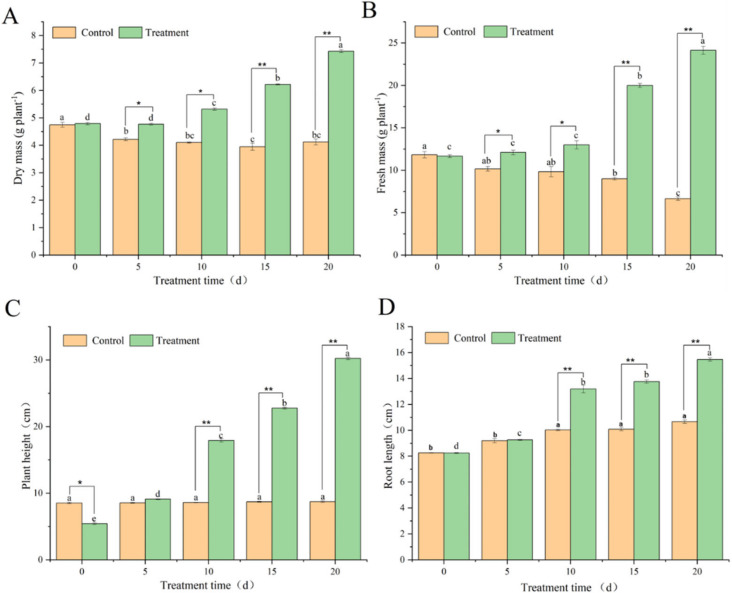
The effect of BY6 strain on the growth of Pdpap poplar seedlings. (**A**): Dry mass; (**B**): Fresh mass; (**C**): Plant height; (**D**): Root length. The graph value is the average of four biological replicates; the error bars represent the standard error of the average (*n* = 4) at each time point; The experiment was carried out in four biological replicates. Different * indicate significant differences between the groups at the same time point (ANOVA, * < 0.05; ** < 0.01), and the different lowercase letters indicate significant differences for the same treatment group at different times (ANOVA, *p* < 0.05).

To analyze the impact of BY6 on the growth of diseased Pdpap poplar seedlings, the dry mass, fresh mass, plant height, and root length of the seedlings were all determined. On the 5th, 10th, 15th, and 20th d of inoculation with the BY6 strain, the dry mass index of the treated group exhibited an upward trend (Figure 4A) and finally reached the maximum value on the 20th day. The infected control group clarified a slight downward trend from 0 to 20 days. The fresh mass of the treated group exhibited an upward trend on the 10th, 15th, and 20th day (Figure 4B) and reached a maximum value on the 20th day, which was 2.07 times higher than that on the 0th day. It revealed a downward trend, reaching a minimum value on the 20th day, which was 1.77 times lower than 0 day. The plant height of the treated group demonstrated an upward trend from 0 to 20 days (Figure 4C), and the difference was significant. It reached a maximum value on the 20th day, which increased by 5.55 times compared to day 0, while the difference between the infected control groups was insignificant. The root length of the treated group demonstrated an upward trend from 0 to 20 days (Figure 4D), reaching a maximum value on the 20th day, 1.87 times higher than that on day 0. The infected control group stabilized after a slight increase from 0 to 10 days. In conclusion, after induction, the BY6 strain promoted the growth of diseased Pdpap poplar seedlings.

### 3.5. Determination of Soluble Protein and Soluble Sugar in Pdpap Poplar after BY6 Induction

**Figure 5:**
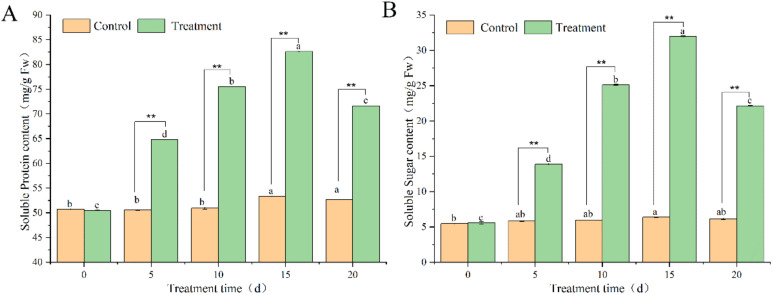
Effects of BY6 on soluble protein (**A**) and sugar (**B**) content in Pdpap poplar leaves. The experiment was carried out in four biological replicates; ** indicate significant differences between the groups at the same time point (ANOVA, ** < 0.01), and the different lowercase letters indicate significant differences for the same treatment group at different times (ANOVA, *p* < 0.05).

The results in Figure 5A show that the soluble protein content in the treated group had an upward trend from 0 to 15 days, reaching a maximum on day 15. Compared with day 0, it increased by 1.63 times, then decreased slightly from the 15th to the 20th day. The infected control group demonstrated a minor fluctuation, and the overall level was stable. (Figure 5B) displays that the soluble sugar content of the treated group demonstrated an overall upward trend from 0 to 15 days and reached the maximum value on day 15, which increased by 5.72 times compared at day 0. A slight decrease was observed from 15 to 20 days, whereas the infected control group stabilized overall.

### 3.6. Determination of Expression Levels of Disease Resistance and Growth-Related Genes in Pdpap Poplar after BY6 Induction

**Figure 6:**
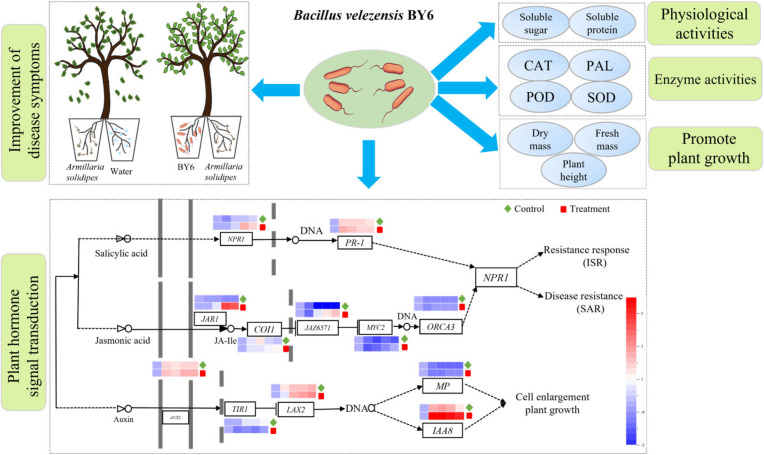
Schematic representation of the growth-promoting and disease-resistant responses of BY6 to diseased Pdpap poplar after induction. SA pathway genes (*NPR1*; *PR-1*). JA pathway genes (*JAR1*; *COI-1*; *JAZ6571*; *MYC-2*; *ORCA-3*). Auxin pathway genes (*AUX1*; *TIR1*; *LAX2*; *MP*; *IAA8*). Color bar: the red part represents genes with positive expression levels, and the blue represents negative expression levels. The roots of Pdpap poplar seedlings were divided into two equal parts (part 1 and part 2), and transplanted solid and dashed arrows indicate direct and indirect activation, respectively. Prohibition sign means prohibition.

The expression of genes related to the signal transduction pathway of disease resistance in Pdpap poplar was determined (Figure 6). The first was the SA pathway illustrateed. The *NPR1* genes expression levels in the BY6-treated group demonstrated an up-regulation trend, reaching a maximum transcriptional peak at 24 h. The expression level was 5.64 times that of the infected control group (Supplementary Table S1); The *PR1* genes-treated and infected control groups revealed an upward-regulated trend, reaching the maximum transcriptional peak at the 6th h.

The second is the JA pathway. The *JAR1* enzyme synthesizes JA-Ile genes treatment group revealed an up-regulation trend, reaching a maximum value at 24 h, while the infected control group demonstrated a down-regulation trend. The *COI* genes expression levels in the BY6-treated group exhibited an upward-regulated trend, reaching the maximum transcriptional peak at 48 h. In contrast, the infected control group was first up-regulated at 0~12 h, and then down-regulated after 12~48 h. The *JAZ6571* genes expression levels in the BY6-treated group displayed an up-regulation trend, reaching the maximum transcriptional peak at 48 h, whereas the infected control group revealed a down-regulation trend. The *MYC-2* genes in the treatment group was up-regulated and reached the maximum transcriptional peak at 48 h, while the infected control group indicated a down-regulation trend. The *ORCA3* genes in the control and genes expression levels in the BY6-treated group revealed a downward trend, and the difference between them was insignificant.

BY6 induces the transcription of Pdpap poplar auxin signaling-related genes. The *AUX1* genes in the control and genes expression levels in the BY6-treated group indicated an upward-regulated trend. The treatment group reached the maximum transcription peak at 24 h, and the infected control group was up-regulated at 0~6 h first. There was no significant change between 6~48 h. The *TIR1* genes in the infected control group indicated an up-regulation trend, reaching the maximum transcriptional peak at 48 h, and the treatment group demonstrated a down-regulation trend. The *LAX2* genes in the control and genes expression levels in the BY6-treated group exhibited the same performance; they indicated an upward-regulated trend and reached the maximum transcription peak at 72 h. In contrast, the *MP* genes in the control and gene expression levels in the BY6-treated group revealed a downward trend. The changing trend of the *IAA8* genes treatment and infected control groups was the same. The maximum transcription peak was reached at the 12th hour, and the maximum transcription peak of the genes expression levels in the BY6-treated group was 1.95 times that of the infected control group. This result indicated that BY6 promoted the expression of genes related to disease resistance signal transduction and the growth pathway in Pdpap poplar.

## 4. Discussion

To further understand the biological control effect of the BY6 strain, its effect of BY6 on the ARR treated disease index and relative control of diseased Pdpap poplar was determined under indoor conditions. The results demonstrate that, compared with the infected control group, the treated disease index of Pdpap poplar root rot disease decreased by 42.02% after inoculation with BY6, and the relative control effect exhibited an upward trend; this confirms that BY6 has a therapeutic effect on the diseased Pdpap poplar and first report of *B. velezensis* BY6 for controlling ARR in Pdpap poplar. *Bacillus* strains are known for their metabolic capability and environmental versatility as well as for their ability to manage bacterial and fungal pathogens infecting plants. In some similar studies, Sarah Hong et al. [23] using *B. velezensis* CE 100 not only increased the fruit yield of strawberries by controlling the fungal diseases (*Macrophomina phaseolina and Fusarium oxysporum f.* sp. *fragariae*) but also through enhancing plant growth. Peng Hong et al. [24] also using *Bacillus amyloliquefaciens* HF-01, SBC control of postharvest decay on citrus while maintaining fruit quality after harvest. The strain BY6 of *B. velezensis* proved its antagonistic potential in controlled, reducing ARR on Pdpap poplar.

It remains unclear whether the symptomatic relief of ARR in Pdpap poplar is related to BY6 induction. To some extent, the interaction between plants and pathogens is a process by which the pathogen hydrolase system of the pathogen associates with the oxidase system of the host plant [25]. It has been reported that oxidase activity levels (POD, PAL, SOD, and CAT) increase or decrease after inoculation with inducers, further enhancing plant resistance to pathogen invasion and expansion [22]. PAL is the rate-limiting enzyme in the phenylpropane metabolic pathway, which regulates the accumulation of the main antibacterial substances. Therefore, it is often used to evaluate plant disease resistance [26]. POD is a terminal oxidase in the metabolic pathway of phenylpropane that strengthens the cell wall by catalyzing the polymerization of phenolic acids and the synthesis of lignin and enhances the resistance of plants to pathogenic infection. Under certain conditions, POD can also have a synergistic effect with PAL and CAT to eliminate the damage caused by excessive reactive oxygen species that are toxic to cells in the host [27]. PAL and POD activities in Pdpap poplar leaves were significantly increased after inoculation with BY6, BY6 induced the activation of Pdpap poplar defense enzymes and reduced the toxicity of pathogenic infection to its cells. *Bacillus* can increase the content of total phenols, flavonoids, and lignin in plants by activating plant PAL and POD enzyme activities [28]. These compounds can directly inhibit spore germination and mycelial growth of pathogenic fungi and the precursors for lignin synthesis. Lignin deposition on cell walls can improve mechanical tissue strength and prevent pathogen invasion. A same plant split into two parts was employed in this study, and the BY6 strain was not in contact with *A. solidipes*, which ruled out the bacteriostatic effect of BY6. Instead, BY6 induced Pdpap poplar cell wall enhancement, which reduced the damage caused by *A. solidipes* to invasion.

Prior studies [29] also revealed that the induction of Pdpap poplar with *B. amyloliquefaciens* AW3 could increase the CAT activity of Pdpap poplar leaves, thus increasing the root rot of Pdpap poplar caused by *Fusarium oxysporum* infection. CAT activity significantly decreased after BY6-induced Pdpap poplar and obtained the opposite results. We speculate that, on the one hand, the changes in various enzyme activities caused by pathogenic bacteria are related to the degree of plant damage. In contrast, BY6 induction alleviated the stimulation of pathogenic bacteria to Pdpap poplar, thus reducing CAT enzyme activity. To confirm the presumed results, we tested reactive oxygen species (ROS) and cell death in Pdpap poplar leaves. As a consequence of trypan blue staining, more necrosis was observed in the leaves of the infected control group. DAB staining also revealed increased H_2_O_2_ accumulation in the leaves of the infected control group. Both indicated that BY6 induction alleviated the pathogenic stimulation of Pdpap poplar and decreased CAT activity. Notably, the prevention and control of diseases by biocontrol bacteria result from multiple mechanisms (such as niche and nutrient competition, secretion of antimicrobial substances, host resistance induction, and colonization ability) [30]. It is also associated with other microbial community structures in plants [27]. These factors affect biological control and the multiple mechanisms involved require further investigation.

In addition to preventing and controlling plant diseases, some biocontrol bacteria have growth-promoting effects [29]. The dry mass of treated groups increased significantly on the 5th, 10th, 15th^,^ and 20th d after inoculation with the BY6 strain, and the fresh mass had the same performance. Dry and fresh mass are important indicators of plant growth status and organic matter accumulation and are indirectly involved in plant disease resistance [31,32]. BY6 can significantly increase the fresh and dry mass of the seedlings after inducing diseased Pdpap poplar seedlings. The results of this study are consistent with those reported by Jin et al. [33]. This study also found that BY6 induction increased the plant height of diseased Pdpap poplar seedlings. The root length of the BY6-treated group revealed an overall increase from 0 to 20 days, while the infected control group indicated no significant increase. We speculate that BY6 could reactivate the expression of auxin-related genes in diseased Pdpap poplar. To test this speculation, verified by qRT-PCR, the results show that the transcription of four auxin-related genes (*AUX1*, *TIR1*, *LAX2,* and *IAA8*) was activated, which confirmed the above speculation. The above results indicate that BY6 secondary induced the growth of diseased Pdpap poplar seedlings, which could have a crucial role in controlling the upper and lower levels.

Soluble sugars and proteins content are critical indicators for measuring a plant’s growth status and quality. They are involved in many metabolic and energy supply processes, and their content is an important indicator for characterizing the physiological activity of plants [34]. Soluble protein and sugar content levels demonstrated an upward trend, suggesting that both increased after BY6 induction. Previous studies have confirmed that soluble sugars and proteins are the main photosynthetic products of higher plants and are also the primary form of carbohydrate metabolism and temporary storage [35]. They also regulate oxidase indicators, and changes in their content can reflect plant health. To verify this relationship, oxidase indicators revealed that, post BY6 induction, this activated SOD, POD, and CAT enzymes (expressed or inhibited) in diseased Pdpap poplar; this indicates that BY6 promotes the production of soluble protein and sugar in the diseased Pdpap poplar, indirectly regulating the activity of oxidase indicators in diseased Pdpap poplar and improving disease resistance. These results are consistent with the reported results for Cf-10-mediated and other leaf mold samples [36].

This study determined whether BY6 triggers defense responses in Pdpap poplar seedlings in the presence of fungal pathogens and offers insights into its molecular basis. The results depicted that the relative expression levels of *NPR1*, *PR1*, *JAR1*, *JAZ6571*, *MYC2,* and *ORCA3* increased after BY6 induction, suggesting that BY6 activated the induced systemic resistance (ISR) of Pdpap poplar seedlings. However, SA and JA signals are antagonistic and cooperative in activating the plant response system for disease resistance [37,38]. It is generally believed that the SA signaling pathway is primarily involved in the resistance response to biotrophic pathogens (obligate parasites), and JA is involved in resistance responses to necrotrophic pathogens [39]. This study found that after the infection of the pathogen *A. solidipes*, BY6 continued to induce the SA and JA pathways. *NPR1*, *PR1*, *JAR1*, *JAZ6571*, *MYC2,* and *ORCA3* genes continued to be expressed, and the expression fold was higher than that of the infected control group under *A. solidipes* infection alone; this suggests that BY6 induced ISR and systemic acquired resistance (SAR) in diseased Pdpap poplar. In some related studies, rhizosphere-promoting bacterium *Bacillus subtilis* PTA-271 is an essential component of the *Arabidopsis thaliana* ISR, and the elicited ISR is JA/ET pathways dependent, and not pathogen-dependent SAR [40]. This study found that probiotic BY6 induced both SAR and ISR of the diseased Pdpap poplar, not a single ISR. It is an interesting phenomenon, and the specific mechanism will be examined in follow-up studies in the future.

## 5. Conclusions

This study used the endophyte *B. velezensis* BY6 to induce the mechanism of growth promotion and disease resistance in diseased Pdpap poplar. The results show that the application of BY6 on Pdpap poplar seedlings resulted in a significant increase in plant growth indicators (dry mass, fresh mass, and plant height), and also observed that the transcription of four auxin-related genes (*AUX1*, *TIR1*, *LAX2,* and *IAA8*) was significantly increased. BY6 revealed a remarkable control effect after the inoculation of diseased Pdpap poplar plants. On the 20th day, the treated disease index of the treatment group was reduced by 49.53%, compared with those of the infected control group. The relative staining area of DAB and Trypan blue decreased by 3.37 and 7.31 times, respectively, and the physiological indicators (soluble sugar and soluble protein) and oxidase indicators (CAT, PAL, POD and SOD) were significantly improved. The expression levels of defense-related genes (*NPR1*, *PR1*, *JAR1*, *JAZ6571*, *MYC2,* and *ORCA3*) in SA and JA signaling pathways were significantly increased. To the best of our knowledge, this is the first report in which BY6 controls ARR during diseased Pdpap poplar progression, involving the molecular mechanisms underlying growth promotion and disease resistance.

## Figures and Tables

**Table 1:** Genes and primers used for the RT-qPCR analysis.

Target Gene	Primer Sequence (5′–3′)	Predicted Product Size (bp)
*PR1*	ACACCACCGTGCAACCTATG	240
	CGAGCAGAGTTCGCCAAACCA	
*NPR1*	GGCCGACGATATTCCCTAGTT	261
	TGCCCTCATAGTTTCTGAGCT	
*AUX1*	CAGTCGGTGCTCTTCGGTCA	230
	GTCCACCTGCCTACGAAGT	
*LAX2*	TCATTCGGCCCTTCACCAAGT	212
	GCCATGATTGCATCACACCTT	
*MP*	GCACATGAACGGCAGGGTTT	213
	ACCTGACCATGACGACACTT	
*TIR1*	GTTGGTACGCAAAGGTAGAGA	235
	GGCTGACCATGCAATACTAGC	
*IAA8*	CGGAGACTGGATGTTGTTGGT	226
	ACCTGAAACCTGATCGTGCTC	
*JAR1*	AGTGGTGACCAAGCGAGGAG	210
	GGATTTGTGCACCTTGCTGTT	
*JAZ6571*	GGAAGCTCATTGGCACAGATG	221
	CCGGAGTGGGTTGTTTGTCTG	
*MYC2*	ACGAAGCGCAATCTGCTGAGT	223
	CCAGGCTCTCAAAGCCGACAT	
*COI*	GAGGTATTGTGGGTGCATGGT	245
	ACGCGAACCTACCCTCGCT	
*ORCA3*	AGAGGATGAGGCAAAGACCCT	231
	TCCGGCTCTCGCGCTTAGT	
*Actin(P)*	AGCTGATCGAATAGCAAG	196
	CTAGAAGCACTCCTGTG	
*β-tubulin (P)*	TACCGAGGCTGAGATAACAT	210
	GGACCCACAACTCATCACAT	

Note: Pathogenesis-related 1 (*PR1*); Non expressor of pathogenesis-related 1 (*NPR1*); Auxin resistant 1 (*AUX1*); Auxin influx carrier protein (*LAX2*); Monopteros (*MP*); Transport inhibitor response protein 1 (*TIR1*); Auxin/indoleacetic acids proteins (*IAA8*); Jasmonate resistant 1 (*JAR1*); Jasmonate ZIM-domain (*JAZ6571*); V-myc avian myelocytomatosis viral oncogene homolog (*MYC-2*); Cytochrome c oxidase I (*COI*); Octadecanoid-derivative responsive Catharanthus AP2-domain protein 3 (*ORCA3*).

## Data Availability

Not applicable.

## Supplementary Materials

The following supporting information can be downloaded at: https://www.mdpi.com/article/10.3390/microorganisms10122472/s1, Table S1: RT-qPCR analysis date.
